# Judging quality of current septic shock definitions and criteria

**DOI:** 10.1186/s13054-015-1164-6

**Published:** 2015-12-25

**Authors:** Manu Shankar-Hari, Guido Bertolini, Frank M. Brunkhorst, Rinaldo Bellomo, Djillali Annane, Clifford S. Deutschman, Mervyn Singer

**Affiliations:** Department of Intensive Care Medicine, 1st Floor, East Wing, St Thomas’ Hospital, Guy’s and St Thomas’ NHS Foundation Trust, Westminster Bridge Road, London, SE1 7EH UK; Division of Asthma, Allergy and Lung Biology, King’s College London, ᅟ, SE1 9RT UK; Laboratory of Clinical Epidemiology and GiViTI Coordinating Centre, IRCCS-Istituto di Ricerche Farmacologiche “Mario Negri”, Villa Camozzi 24020 Ranica (Bergamo), Italy; Paul-Martini-Research Group for Clinical Sepsis Research, Center for Clinical Studies, Jena University Hospital, Salvador-Allende-Platz 29, Jena, 07737 Germany; Department of Intensive Care and Medicine, Austin Health, Heidelberg, Melbourne, VIC 3084 Australia; Department of Intensive Care Medicine, Hôpital Raymond Poincaré (AP-HP), Laboratory of Cell Death, Inflammation & Infection, UMR1173 University of Versailles SQY & INSERM, 92380 Garches, France; Departments of Pediatrics and Molecular Medicine, Hofstra-North Shore-Long Island Jewish-Hofstra School of Medicine, New Hyde Park, NY 11040 USA; Feinstein Institute for Medical Research, Manhasset, NY 11030 USA; Bloomsbury Institute of Intensive Care Medicine, University College London, London, WC1E 6BT UK

## Abstract

Septic shock definitions are being revisited. We assess the *feasibility*, *reliability*, and *validity* characteristics of the current definitions and criteria of septic shock. Septic shock is conceptualised as cardiovascular dysfunction, tissue perfusion and cellular abnormalities caused by infection. Currently, for feasibility, septic shock is identified at the bedside by using *either* hypotension *or* a proxy for tissue perfusion/cellular abnormalities (e.g., hyperlactatemia). We propose that *concurrent* presence of cardiovascular dysfunction *and* perfusion/cellular abnormalities could improve validity of septic shock diagnosis, as we are more likely to identify a patient population with all elements of the illness concept. This epidemiological refinement should not affect clinical care and may aid study design to identify illness-specific biomarkers and interventions.

## Introduction

The illness concept, with its description (*definition*), clinical symptoms, signs and laboratory tests (*criteria*), and consequences (*outcome*), inform clinical reasoning and diagnosis. Diagnostic certainty is important for patients, clinicians and researchers as it helps to identify risk factors, select optimal treatment, devise new therapies, and prognosticate. In this context, septic shock is a *clinical syndrome* with protean clinical manifestations and biochemical abnormalities but with no universally accepted gold standard for diagnosis [1]. In the absence of a gold standard test, the clinician-determined probability of the illness, the definition and the corresponding criteria are fundamental to diagnosing syndromes. After outlining the septic shock illness concept, this article evaluates how bedside operationalization of current definitions of septic shock definitions and their corresponding clinical criteria influence the *validity*, *reliability*, and *feasibility* attributes [2].

### Illness concept

**Fig. 1:**
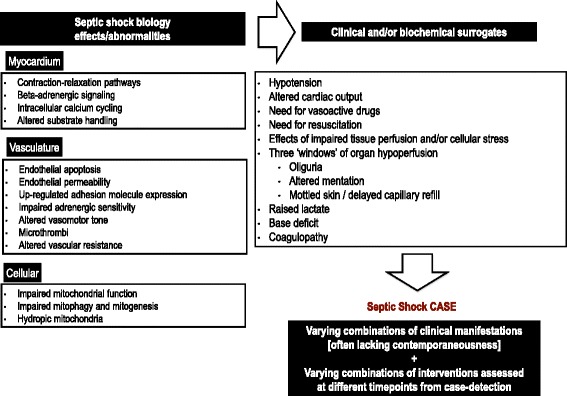
Simplified overview of septic shock biology

Infection triggers a series of host responses, dysregulation of which results in organ dysfunction (sepsis) [3–5]. Septic shock, the most severe form of sepsis, is highly complex, with concurrent myocardial, vascular (macro- and micro-), tissue perfusion and cellular level abnormalities. Myocardial abnormalities include both cardiomyocyte injury [4] and structural myocardial dysfunction [6, 7]. Vascular abnormalities affect the endothelium, sub-endothelial tissues, microcirculation and vasomotor tone. These alter systemic vascular resistance, increase endothelial permeability, impair tissue oxygen delivery (tissue hypoxia) and utilization (cellular dysoxia), and modify cellular metabolism (Fig. 1) [8–15].

### Current definitions and criteria for septic shock

The 1992 and 2001 Sepsis Consensus Conference definitions [16, 17] and the definition used by the Surviving Sepsis Campaign (SSC) guidelines [18] currently provide the most-cited case definitions and criteria for making a diagnosis of septic shock. Yet all differ for both definition and criteria (e.g., use of different cutoffs for hypotension) and variable use of markers of impaired tissue perfusion (e.g., raised lactate, base deficit). There is also an implicit reliance upon clinician-driven interventions such as fluid therapy or inotropes. Thus, there are potentially varying combinations of clinical criteria and differences in the interventional targets used to diagnose septic shock. This heterogeneity highlights the limitations of current operationalization methods [19–21].

## Judging quality attributes of septic shock definitions and criteria

### Validity

Validity is the ability to capture what the investigator truly seeks to measure. Validity assessments can be either qualitative (face validity, content validity) or quantitative (criterion validity, construct validity).

#### Face validity

“Face validity” refers to whether patients identified by the criteria appear to match the illness concept at face value. At present, hypotension is a core element of the criteria identifying septic shock. However, the presence of hypotension in the context of infection does not necessarily define septic shock. Hypotension in an infected patient could be related to pre-illness medications (e.g., antihypertensive), comorbidities (e.g., heart failure) or concurrent interventions (e.g., sedatives) or a combination of these. In other words, we remain uncertain whether hypotension as the only criterion truly represents septic shock, especially when used without caveats to address potential confounders (e.g., medications or heart failure). Furthermore, we cannot quantify this uncertainty, as there is no gold standard test for septic shock.

#### Content validity

Content validity assesses whether the clinical criteria encompass all components of the illness. For septic shock, these components are cardiovascular dysfunction, hypoperfusion and cellular abnormalities. We highlighted above that hypotension alone is an incomplete proxy for cardiovascular dysfunction to define septic shock. Equally, imperfect proxies of tissue hypoperfusion such as base deficit, confusion or oliguria are inadequate in isolation to define septic shock [16]. Similarly, we currently lack a reliable proxy to measure the complex cellular abnormalities that occur in septic shock. Hyperlactatemia is often used as a proxy; however, this is usually found in all types of shock [8] and the serum level is variably related to multiple confounders, including the rate of tissue utilization (lactate clearance), accelerated β-adrenergic-driven aerobic glycolysis, liver dysfunction and co-existing anaerobic glycolysis [11, 22–25].

Given the lack of a gold standard diagnostic test for septic shock, the performance characteristics of these variables (sensitivity, specificity, and predictive values) could be based on prognosis (e.g., short-term mortality), with or without a clinical reclassification risk assessment or blinded clinician adjudication [26]. If we alter the existing clinical criteria, the reclassification risk refers to the proportion of patients reclassified into new risk-of-death categories [26]. Levy et al., using the SSC dataset, gave a simple descriptive demonstration of this concept by using lactate as an additional marker for septic shock in a cohort of patients with an overall mortality of 38.4 % [27]. The authors reported categories of shock as lactate of more than 4 mmol/l only (29.9 % mortality), vasopressor use only (36.7 % mortality), or a combination of lactate of more than 4 mmol/l plus vasopressor use (46.1 % mortality). A similar exercise conducted on data collected from English intensive care units (ICUs) reported mortality rates of 26.2 % for patients with an isolated lactate level of more than 4 mmol/l, 31.4 % for refractory hypotension only, and 55.5 % for the combination [28]. Kaukonen et al. recently evaluated the performance characteristics of the four systemic inflammatory response syndrome (SIRS) criteria in identifying sepsis [29]. They highlighted that 12 % of patients admitted to ICUs with organ dysfunction consequent to presumed infection were SIRS-negative (i.e., had fewer than two criteria of SIRS). Thus, if sepsis criteria are developed without requiring SIRS variables, different risk categories within patient populations may be identified (i.e., reclassification).

While cardiovascular organ dysfunction has long been central to the definition of septic shock, the criteria used to identify it remain variable. Levy et al. [17] specified the use of either Sepsis Organ Failure Assessment (SOFA) or multiple organ dysfunction score (MODS) to quantify organ dysfunction in patients with severe sepsis [30, 31]. However, these two scoring systems differ, particularly in terms of cardiovascular dysfunction criteria. The SOFA score uses both mean arterial pressure and the dose of vasoactive drugs being administered. By contrast, the MODS is solely physiology-based, using a pressure-adjusted heart rate parameter derived from heart rate, central venous pressure and mean arterial pressure to reflect fluid-unresponsive hypotension. Under the MODS scoring system, organ dysfunction is a continuum with no category differentiating dysfunction from failure [30–32]. Cardiovascular dysfunction is quantified by a continuous variable derived by using regression analyses to define the variables and their weights. In contrast, SOFA, which was developed by using expert opinion, views cardiovascular dysfunction as occurring in discrete steps, with a score of 1 or 2 being coded as organ “dysfunction” and 3 or 4 coded as organ “failure”. Thus, the cutoffs and relative weights of variables within these scores are unlikely to stratify cardiovascular dysfunction similarly [33].

#### Criterion and construct validity

Criterion validity encompasses both concurrent and predictive validities. Concurrent validity refers to the ability of the definition and criteria to discriminate groups, whereas predictive validity is the ability to predict (future) outcomes. A closely related concept is construct validity; this refers to an assessment of how well the definitions are converted into measurable criteria to identify septic shock in clinical practice.

There are many examples in the literature of poor criterion validity for current definitions and criteria of septic shock. For example, a cohort study of nearly 8000 patients defined by the 1992 criteria as having septic shock [16] reported an overall crude hospital mortality of 52.4 % [20]. However, mortality ranged from 21.1 to 84.5 % when stratified on anatomical site of infection, and this variation persisted despite adjustments for confounders such as age, comorbidities and organism type. Given that cardiovascular dysfunction is the core criterion used for diagnosis, the predictive validity of current definitions of septic shock is weak, being heavily dependent on how it is operationalized at the bedside [19].

Outcome is also affected by how the individual components of septic shock (cardiovascular dysfunction, hypoperfusion and cellular abnormalities) are determined. Mortality in a single dataset varied from 45 to 60 % depending on what diagnostic criteria were applied [34]. Similarly, hospital mortality rates in septic shock patients admitted to ICUs in Australia and New Zealand (171 ICUs; *n* = 6757) (ANZICS data, R. Bellomo, personal communication) and Italy (221 ICUs; *n* = 4959) (GiViTI data, Italian ICU registry; Margherita project, G. Bertolini, personal communication) during 2012 were reported as 22 and 57.9 %, respectively. The Australasian case definition was predicated upon an APACHE III (Acute Physiology and Chronic Health Evaluation III) diagnosis coding of infection plus recording of either a decrease in mean blood pressure of less than 65 mmHg or systolic blood pressure of less than 90 mmHg at any time within the first 24 h of admission. In contrast, the Italian criteria used those provided by the 2001 Consensus definitions [17]. Even when data were extracted from representative national datasets by using the International Classification of Diseases coding system, the hospital mortality using the septic shock code was much higher in Germany (60.5 % in 2011) [35] than in the USA (42.1 % between 2004 and 2009) [21]. Are outcomes in Germany much worse, or do the differences merely reflect coding practices or variable patient pathways (e.g., transfer to post-acute care hospitals) [36]?

**Fig. 2:**
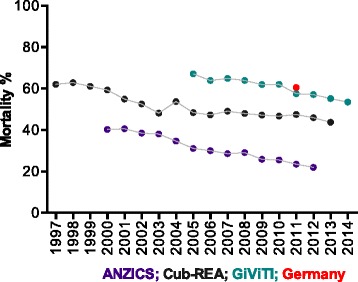
Secular trends in septic shock mortality. Cub-REA Data were provided by Philippe Aegerter, Bertrand Guidet and D. Annane for the Cub-REA network, which used International Classification of Diseases (ICD)-9 and ICD-10 codes and the Bone definition [16]. GiViTI data were provided by Bertolini et al. using 2001 Consensus Definitions [16, 17]. ANZICS data, provided by Bellomo et al. (personal communications), used hypotension as the definition for septic shock. Brunkhorst et al. (personal communications) provided German data for 2011, using ICD codes for the diagnosis of septic shock

Secular trends in septic shock outcomes are depicted in Fig. 2. Among other explanations [37], this improving trend in mortality may also represent an enhanced detection of a less severe “septic shock” population using hypotension alone as a criterion (i.e., stage migration, or the Will Rogers phenomenon [38]).

### Reliability

Reliability refers to agreement between observers and by the same observer during repeated measurements (i.e., consistency and reproducibility). With septic shock, some measurements, such as blood pressure and lactate levels, are likely to have reasonably high inter-observer, intra-observer and intra-subject reliability subject to certain caveats and exclusion of methodological errors. On the other hand, adequacy of fluid resuscitation and initiation of vasopressor therapy are far more subjective because of a lack of consensus on triggers and end-points. Potentially, we can test the reliability attributes of these variables (e.g., consistency within and between observers) by using test-retest reliability and the related kappa statistic.

### Feasibility

Feasibility is a composite concept that depends on the purpose of the diagnosis; it is a compromise between validity and reliability [2]. For a high-mortality condition such as septic shock, ease of detection is key. The SSC guidelines have increased feasibility by emphasizing cardiovascular dysfunction criteria for the diagnosis of septic shock [18]. However, relaxing diagnostic criteria will almost certainly increase the rate of false-positive diagnoses. Conversely, if the complexity and number of criteria to be met for diagnosis are increased to improve validity, then feasibility will almost certainly be reduced. For example, the incidence of septic shock was halved from 9.1 to 4.4 % when liberal criteria (i.e., refractory hypotension) were replaced with restrictive criteria (i.e., refractory hypotension with non-cardiovascular dysfunction) [34].

## How could we address these challenges?

The above discussion highlights variability in the criteria used to identify septic shock in published research. The European Society of Intensive Care Medicine-Society of Critical Care Medicine (ESICM-SCCM) Task Force on new definitions of sepsis is currently undertaking a systematic review of the literature to explore how these septic shock definitions and criteria are operationalized. Observational studies reporting the incidence and outcomes of septic shock are ideal target publications as randomized controlled trials often have trial-specific criteria. The second step in this process is to generate agreement on the updated illness concept which should reflect all three domains of biology (i.e., cardiovascular dysfunction, cellular abnormalities and evidence of impaired tissue perfusion). Based on this illness concept and by applying the principle of parsimony, the minimum number of bedside variables that have face and content validity could be determined. Inter-observer reliability of septic shock case detection could be improved by using a single validated criterion for cardiovascular dysfunction (e.g., need for vasopressor therapy to maintain target blood pressure) and a proxy for likely cellular and impaired tissue perfusion abnormality (e.g., lactate). These steps could be achieved with qualitative research methods, such as a Delphi process [39]. An important research question in this context is whether septic shock illness criteria should have predictive validity [19]. If we believe that septic shock is the most severe form of sepsis, then an argument could be made for having predictive validity as a characteristic of any updated septic shock criteria. This research question could be addressed by developing a parsimonious model using combinations and cutoffs of the variables for outcomes such as short-term mortality.

## Conclusions

Septic shock conceptually comprises an illness with new onset or worsening cardiovascular dysfunction, impaired tissue perfusion and cellular abnormalities caused by infection. Individually, none of these abnormalities truly reflects the complex illness concept but they may do so in combination, potentially improving the validity and reliability of a diagnosis of septic shock. As there are no specific treatments for this condition, this epidemiological refinement will not affect clinical care and may aid the design of studies that can identify illness-specific biomarkers and interventions.

## Abbreviations

ICU: Intensive care unit
MODS: Multiple organ dysfunction score
SIRS: Systemic inflammatory response syndrome
SOFA: Sepsis Organ Failure Assessment
SSC: Surviving Sepsis Campaign
